# Altered Effective Connectivity in Schizophrenic Patients With Auditory Verbal Hallucinations: A Resting-State fMRI Study With Granger Causality Analysis

**DOI:** 10.3389/fpsyt.2020.00575

**Published:** 2020-06-24

**Authors:** Jie Gao, Dongsheng Zhang, Lei Wang, Wei Wang, Yajuan Fan, Min Tang, Xin Zhang, Xiaoyan Lei, Yarong Wang, Jian Yang, Xiaoling Zhang

**Affiliations:** ^1^Department of MRI, Shaanxi Provincial People’s Hospital, Xi’an, China; ^2^Department of Radiology, The First Affiliated Hospital of Xi’an Jiaotong University, Xi’an, China; ^3^Department of Psychiatry, The First Affiliated Hospital of Xi’an Jiaotong University, Xi’an, China

**Keywords:** schizophrenia, auditory verbal hallucination, magnetic resonance imaging, resting state, effective connectivity

## Abstract

**Purpose:**

Auditory verbal hallucinations (AVH) are among the most common and prominent symptoms of schizophrenia. Although abnormal functional connectivity associated with AVH has been reported in multiple regions, the changes in information flow remain unclear. In this study, we aimed to elucidate causal influences related to AVH in key regions of auditory, language, and memory networks, by using Granger causality analysis (GCA).

**Patients and Methods:**

Eighteen patients with schizophrenia with AVH and eighteen matched patients without AVH who received resting-state fMRI scans were enrolled in the study. The bilateral superior temporal gyrus (STG), Broca’s area, Wernicke’s area, putamen, and hippocampus were selected as regions of interest.

**Results:**

Granger causality (GC) increased from Broca’s area to the left STG, and decreased from the right homolog of Wernicke’s area to the right homolog of Broca’s area, and from the right STG to the right hippocampus in the AVH group compared with the non-AVH group. Correlation analysis showed that the normalized GC ratios from the left STG to Broca’s area, from the left STG to the right homolog of Broca’s area, and from the right STG to the right homolog of Broca’s area were negatively correlated with severity of AVH, and the normalized GC ratios from Broca’s area to the left hippocampus and from Broca’s area to the right STG were positively correlated with severity of AVH.

**Conclusion:**

Our findings indicate a causal influence of pivotal regions involving the auditory, language, and memory networks in schizophrenia with AVH, which provide a deeper understanding of the neural mechanisms underlying AVH.

## Introduction

Auditory verbal hallucinations (AVH) are the most prominent and burdensome symptoms of schizophrenia, and comprise sensory experiences wherein voices are heard without a causative external stimulus. The prevalence of AVH exceeds 60% in patients with schizophrenia worldwide (1, 2). In 30% of patients with AVH, the hallucinations are not affected by clinical treatments (3), and cause functional disability and therefore lower quality of life. Clinical options to treat such patients are currently limited. Since the neurobiological mechanisms involved in AVH remain unclear; insights into the pathophysiology of AVH could facilitate the development of novel strategies to treat patients who do not respond to currently used medications.

The mechanism by which AVH occurs spontaneously from the brain’s intrinsic activity is of great clinical interest. Resting-state functional magnetic resonance imaging (fMRI) can show spontaneous brain activity *in vivo*, and can therefore provide invaluable insights into the psychopathology of AVH. Based on time series derived from resting state fMRI data, functional connectivity (FC) analysis can be used to calculate the temporal correlations between a blood-oxygenation-level dependent in any two regions, offering an effective method to study alterations in brain connectivity within multiple brain networks associated with AVH in schizophrenia.

In previous models including the “memory intrusion”, “self-monitoring”, “two hit bottom-up and top-down”, and hybrid models, language, auditory, and memory brain networks were closely associated with AVH in patients with schizophrenia (4). The superior temporal gyrus (STG) (auditory cortex), Broca’s area (speech production), Wernicke’s area (language comprehension), hippocampus (memory retrieval), and putamen (initiating language representations) are the key areas responsible for AVH in schizophrenia, and showed functional alterations in previous brain imaging studies (5–18). Although aberrant FC associated with AVH has been reported in these regions in many resting-state fMRI studies (6, 7, 15, 16, 19–25), the interactions between these regions have not been described completely. Furthermore, the information flow within these networks has not been completely characterized.

Effective connectivity (EC) analyses using data on time-lagged relationships between cerebral regions, offers additional information on the directionality of information flow within brain networks (26, 27). Granger causality analysis (GCA) is a special method used to study EC, and could yield deductive networks based on hypothetical seed regions without prior knowledge. This model is simple, has low computational complexity, and is not limited by the number of brain regions studied. It is therefore an efficient and convenient method for analyzing fMRI data on resting-state functional organization in various healthy and diseased brain networks (28–30). To our knowledge, this method has not been used to study the information flow within the aforementioned regions in resting-state fMRI studies.

In this study, we used the GCA method on resting-state fMRI data to determine the patterns of effective connections between the bilateral STG, Broca’s area, Wernicke’s area, hippocampus, and putamen in schizophrenia patients with and without AVH, in order to obtain novel imaging evidence for the elucidation of the neural mechanisms underlying AVH. We first used GCA to study the differences in effective connections between schizophrenia patients with and without AVH to determine the direction of connection changes. We then performed correlation analysis between changes in causal influence and severity of hallucination symptoms to identify the functional disturbances caused by AVH.

## Materials and Methods

### Subjects

This study was conducted in accordance with the Declaration of Helsinki. The experimental protocol was approved by the Ethics Committee of Shaanxi Provincial People’s Hospital, and written parental consent was obtained from all participants.

Study participants were recruited from the in-patient ward, and met the following criteria: 1) diagnosed with schizophrenia/schizoaffective disorder by using the Structured Clinical Interview of the DSM-IV (SCID); 2) with the Positive and Negative Syndrome Scale (PANSS) assessment; 3) Han Chinese in origin; 4) right-handed; 5) between 18 and 40 years old; 6) received conventional MRI and resting-state fMRI scans with available images. Exclusion criteria were as follows: 1) history of other psychotic disorders; 2) substance abuse or dependence; 3) severe medical disorders; 4) traumatic brain injury; 5) electroconvulsive therapy within the past 6 months; 6) intellectual disability or neurological impairment. Patients who presented with AVHs at least once a day for the past four weeks, with a PANSS P3 score ≥4 were assigned to the AVH group, and patients who did not experience hallucinations or had a P3 score of ≤3 belonged to the non-AVH group (31, 32).

All clinical data were double reviewed by a senior researcher, who verified the credibility of the patient statements. Information of usage of antipsychotics (name, dosage, and duration of drugs used before MRI scanning) was also collected, and the converted antipsychotic doses to chlorpromazine (CPZ) milligram equivalent units were calculated. No significant correlation was found between granger causality (GC) and illness duration or CPZ equivalent in either AVH group or non-AVH group; thus, they were not taken as covariates in further statistics.

### MRI Acquisition

All MRI data were performed within 3 days after the PANSS. Conventional MRI and resting-state fMRI were acquired on a 3.0T scanner (Signa HDxt, General Electric Medical System, Milwaukee, WI, USA) with an eight-channel phase array radio-frequency head coil. Head motion was minimized by positioned with restraining foam pads. Earplugs were used to decrease the effects of scanner noise. The total scan time was <20 min. Three-dimensional fast-spoiled gradient-recalled echo T1-weighted images (repetition time/echo time, 10/4.6–14 ms) and fast-spin echo T2-weighted images (repetition time/echo time, 4,200/116.4–124.0 ms) were obtained. Before fMRI scanning, the patients were instructed to stay awake, relax, keep their eyes closed, and refrain from moving. Blood oxygen level-dependent (BOLD) fMRI was acquired using the following parameters: 185 volumes, echo time = 30 ms, repetition time = 2000 ms, flip angle = 90°, 40 axial slices, slice thickness = 4 mm; field of view, 240 × 240 mm^2^; matrix = 64×64. The acquisition time was 6 min and 30 s.

### Data Preprocessing

Preprocessing of fMRI data was performed using SPM12 software (http://www.fil.ion.ucl.ac.uk/spm). Functional images were subjected to slice-timing correction and were then realigned to the first volume to correct for head motions. The data with head movement > 1.5 mm and/or rotation angle >1.5° were excluded. The realigned images were then spatially normalized to the MNI EPI template and resampled to a voxel size of 3 × 3 × 3 mm^3^. A band-pass filter between 0.01 and 0.08 Hz was subsequently applied to the data to remove the effects of very-low-frequency drift and high-frequency noise. Finally, spatial smoothing was applied using a 6-mm full-width-at-half-maximum Gaussian kernel. The head-motion parameters, white-matter signals, and cerebrospinal-fluid-signals were regressed out of the BOLD signals.

### Regions of Interest (ROIs) Selection

The bilateral STG, Broca’s area, Wernicke’s area, putamen, and hippocampus were selected as the ROIs. Masks representing each of the ROIs were created by using the Wake Forest University PickAtlas tool with TD-ICBM Human Atlas (TD Brodmann) (33). The fMRI time courses of all the voxels located within the masks for each ROI were extracted.

### Granger Causality Analysis

GCA was performed using REST-GCA software (http://www.restfmri.net). By applying an order 2 vector auto-regression model, for any two ROI time series x(t) and y(t), the time domain pairwise GCA components from x(t) to y(t) (F_x→y_) and from y(t) to x(t) (F_y→x_) were calculated respectively. Pairwise Granger causal connectivity indicates that neuronal activity of one region is predictive of activity occurring in another region. Causal influence was normalized using the following computing method (34):

$$R_{\text{x}\to \text{y}}=\left(F_{\text{x}\to \text{y}}-F_{\text{y}\to \text{x}}\right)/\left(F_{\text{x}\to \text{y}}+F_{\text{y}\to \text{x}}\right)$$

R_x→y_ is the ratio describing the relative strength and directionality of the causal influences between x and y. A positive R_x→y_ with a larger absolute value indicates stronger causal influence from x to y, while a negative R_x→y_ with a larger absolute value denotes stronger causal influence from y to x. Changes in R_x→y_ were calculated by analyzing the differences between the AVH and non-AVH groups.

### Correlation Analysis

The association between changes in causal influence and hallucination were assessed using correlation analyses performed between the normalized R_x→y_ ratios of the pairwise ROIs and the PANSS P3 score. The Bonferroni correction was applied to correct for multiple comparisons, and *P* < 0.005 (0.05/10) was considered statistically significant.

### Statistical Analysis

Statistical analysis was performed on SPSS 17.0 (SPSS, Chicago, IL, USA). The measurement data of normal distribution were presented as means ± standard deviations, and categorical data as frequencies and percentages. The two sample *t*-test or χ^2^ test, were used to compare demographic and clinical data between the AVH and non-AVH groups. All statistical tests were two-tailed, and *P* ≤ 0.05 was considered significant.

## Results

### Subjects

This study included 18 patients with AVH and 18 without AVH. The AVH and non-AVH groups did not show signiﬁcant differences in age, gender, education, smoking, drinking, illness duration, proportion of first episode patients, CPZ equivalent dose, total, negative and general symptom severity PANSS score. Positive PANSS score and PANSS P3 score were significantly higher in the AVH group than in the non-AVH group (**Table 1**).

**Table 1:** Demographic and clinical characteristics of AVH and non-AVH groups.

Items	AVH group	Non-AVH group	*P*
Number	18	18	/
Age (years)	24.33 ± 6.16	24.89 ± 6.73	0.81
Gender (female/%)	11(61.11)	10(55.56)	0.74
right handness (R/L)	18/0	18/0	/
Education level (years)	12.33 ± 3.65	11.89 ± 3.39	0.71
Illness duration (months)	38.78 ± 47.55	14.28 ± 18.48	0.05
Smoking (yes/%)	0/0.00	3/16.67	0.23
Drinking (yes/%)	1/5.56	0/0.00	1.00
First episode patients (yes/%)	9/50.00	10/55.56	0.74
PANSS total score	89.28 ± 19.06	77.89 ± 17.20	0.07
PANSS positive score	28.72 ± 5.28	22.56 ± 3.94	<0.001
PANSS negative score	21.67 ± 9.29	21.56 ± 6.56	0.97
PANSS general psychopathology score	17.94 ± 6.33	15.94 ± 5.10	0.30
P3 score	5.06 ± 0.73	1.89 ± 0.90	<0.001
CPZ	328.61 ± 141.44	283.33 ± 140.46	0.34

AVH, auditory verbal hallucinations; PANSS, Positive and Negative Syndrome Scale; CPZ, chlorpromazine.

### GCA Results

Compared with the non-AVH group, AVH group showed an increase in GC from the right homolog of Broca’s area to the left STG, and a decrease from the right STG to the right hippocampus, from the left putamen to the right hippocampus, from the right putamen to the right hippocampus, and from the right putamen to Broca’s area (**Tables 2** and **3**).

**Table 2:** Comparisons of the pairwise GC values (F_x→y_) between AVH and non-AVH groups.

F_x→y_	AVH group	Non-AVH group	T	*P*
STG.R - Hipp.R	0.95	1.65	−2.26	0.03*
Putmen.L - Hipp.R	0.93	1.94	−2.74	0.01*

L, left; R, right; *P < 0.05.

GC, granger causality; AVH, auditory verbal hallucinations; Hipp, hippocampus; STG, superior temporal gyrus.

**Table 3:** Comparisons of the pairwise GC values (F_y→x_) between AVH and non-AVH groups.

F_y→x_	AVH group	Non-AVH group	T	*P*
Hipp.R - Putmen.R	0.94	1.78	−2.46	0.02*
STG.L - Broca.R	2.81	1.64	2.57	0.02*
Broca.L - Putmen.R	1.25	2.23	−2.39	0.02*

L, left; R, right; *P < 0.05.

GC, granger causality; AVH, auditory verbal hallucinations; Hipp, hippocampus; STG, superior temporal gyrus; Broca, Broca’s area.

**Figure 1:**
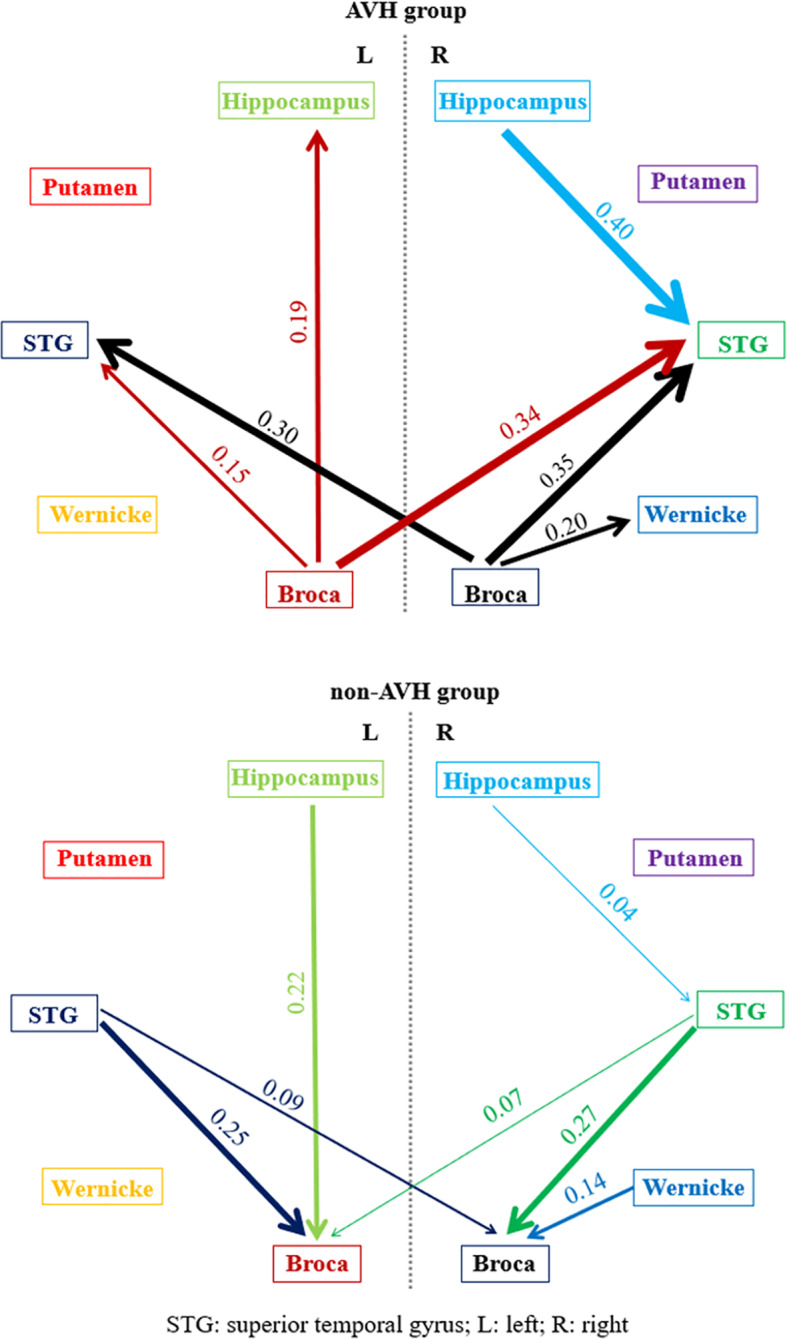
The pairwise normalized ratios of GC values (R_x→y_) in AVH and non-AVH groups. The thickness of lines with marked numbers indicated the strength of granger causality. The different color of lines corresponded to different brain regions.

The normalized ratios of GC from the left STG to Broca’s area and its right homolog, from the right STG to the right homolog of Broca’s area, and from the right STG to the right hippocampus were significantly lower in the AVH group than in the non-AVH group. The bi-directional causal values (F_x→y_ and F_y→x_) explained the results caused by increased GC from Broca’s area and its right homolog to the left STG and from the right homolog of Broca’s area to the right STG, and the decreased GC from the right STG to the right hippocampus (**Table 4** and **Figure 1**).

**Table 4:** Comparisons of the pairwise normalized ratios of GC values (R_x→y_) between AVH and non-AVH groups.

Pairwise ROIs	R_x→y_			F_x→y_			F_y→x_		
	AVH group	Non-AVH group	*P*	AVH group	Non-AVH group	*P*	AVH group	Non-AVH group	*P*
STG.L - Broca.L	−0.15	0.25	0.03*	2.09	1.91	0.74	2.56	1.45	0.06
STG.L - Broca.R	−0.3	0.09	0.04*	2.03	2.16	0.81	2.81	1.64	0.02*
STG.R - Broca.R	−0.35	0.27	0.00**	1.86	2.24	0.38	2.64	1.79	0.06
STG.R - Hipp.R	−0.4	−0.04	0.04*	0.95	1.65	0.03*	1.49	1.93	0.25
Broca.R - Wernicke.R	0.2	−0.14	0.03*	1.93	1.89	0.92	1.58	2.16	0.19
Broca.L - Hipp.L	0.19	−0.22	0.04*	1.35	1.55	0.64	1.01	1.58	0.13
Broca.L - STG.R	0.34	−0.07	0.02*	2.22	1.33	0.05	1.66	1.65	0.99

L, left; R, right; *P < 0.05; **P < 0.001.

GC, granger causality; AVH, auditory verbal hallucinations; Hipp, hippocampus; STG, superior temporal gyrus; Broca, Broca’s area; Wernicke, Wernicke’s area.

The normalized ratios of Granger causality from the right homolog of Broca’s area to the right homolog of Wernicke’s area, from Broca’s area to the left hippocampus, and from Broca’s area to the STG were significantly increased in the AVH group (**Table 4** and **Figure 1**). Bi-directional causal values (F_x→y_ and F_y→x_), indicated that these differences were mainly due to the decreased Granger causality from the right homolog of Wernicke’s area to the right homolog of Broca’s area and from the left hippocampus to Broca’s area, and increased Granger causality from Broca’s area to the STG (**Table 4** and **Figure 1**).

### Correlation Analysis

The normalized ratios of Granger causality from the left STG to Broca’s area, from the left STG to the right homolog of Broca’s area, and from the right STG to the right homolog of Broca’s area were negatively correlated with PANSS P3 score, and the normalized ratios of Granger causality from Broca’s area to the left hippocampus and from Broca’s area to the right STG were positively correlated with PANSS P3 score (**Table 5**). However, after the Bonferroni correction, statistically significant correlations were only observed between normalized ratios of Granger causality from the right STG to the right homolog of Broca’s area and from Broca’s area to the right STG and PANSS P3 score.

**Table 5:** Correlations between pairwise normalized ratios of GC values and PANSS P3 score.

Pairwise ROIs	R	*P*
STG.L - Broca.L	−0.419*	0.026
STG.L - Broca.R	−0.441*	0.019
STG.R - Broca.R	−0.586^#^	0.001
Broca.L -STG.R	0.523^#^	0.004
Broca.L - Hipp.L	0.382*	0.045
STG.R - Hipp.R	−0.334	0.082
Broca.R - Wernicke.R	0.147	0.456

L, left; R, right; *P < 0.05; ^#^P < 0.005.

GC, granger causality; PANSS, Positive and Negative Syndrome Scale; Hipp, hippocampus; STG, superior temporal gyrus; Broca, Broca’s area; Wernicke, Wernicke’s area.

## Discussion

GCA, an effective connection analysis method, is used to examine the directional interaction and influence between brain regions by calculating the information between two time series. In recent years, GCA has been widely applied in the field of neurocognitive science. Studying alterations in interactive causal influences (driving forces) in brain regions involved in schizophrenia-related hallucinations could improve our understanding of the underlying neurobiological substrates. In this study, we found significant differences in effective connections related to auditory, speech, and memory circuits, involving the STG, Wernicke’s area, Broca’s area, and hippocampus, in schizophrenia patients with and without AVH by using GCA. These abnormal functional connections could contribute causally to the onset of auditory hallucinations.

### The Auditory Processing Circuit

The STG contains the auditory cortex, which is responsible for the perception and processing of sounds. Broca’s area is closely associated with the auditory cortex. Previous resting state fMRI studies suggested that abnormal connectivity between Broca’s area and the auditory cortex could be responsible for AVH (8, 24, 35). However, evidence of resting connectivity between these two areas is unclear. Sommer et al. (8) reported reduced synchronization between the left STG and inferior frontal gyrus (IFG, close to Broca’s area) in patients with chronic AVH (8). Hoffman et al. (35) studied the time course of AVH and FC at the various stages of hallucinations (35), and found an increased coupling just prior to hallucinations between the left IFG and right temporal areas (including the STG and middle temporal gyrus). Diederen et al. (24) also observed elevated connectivity between the left STG and right IFG (24). Thus, the change in connection strength in the two regions is not clear, and possibly not uniform in all patients with schizophrenia. Furthermore, the directionality of abnormal connections related to auditory and language processing regions remains unclear.

Our results from the GCA provide information on the direction of the effective connection between the STG and Broca’s area. We observed increased Granger causality from Broca’s area and Broca’s homolog to the bilateral STG in the AVH group, which was also associated with severity of hallucination. To date, only Baojuan et al. (36) have studied the interaction between the auditory cortex and Broca’s area in a dynamic causal model. They reported that a positive correlation was observed between the strength of EC from Broca’s area to the auditory cortex and AVH severity, which is consistent with our results. The increased effective connection from the auditory cortex to Broca’s area was likely a compensatory change due to the decreased connection from Wernicke’s area to Broca’s area (7), which was also verified in our study. In addition, we hypothesize that AVHs are derived from auditory cortical activity, and spontaneous activity is very likely caused by the intrusion of internal auditory signals from Broca’s area.

### The Speech Processing Circuit

Our results showed decreased Granger causality from the right homolog of Wernicke’s area to the right homolog of Broca’s area. Broca’s area is involved in the production of language, and Wernicke’s area is involved in the comprehension of written and spoken language. Although the left hemisphere is widely considered the site of speech processing, studies in patients who suffered strokes indicate that the right hemisphere is also capable of basic language production (37, 38). Further studies indicated that the right homologs of Broca’s and Wernicke’s areas have prominent roles in the processing of emotional information and spoken language tone (39, 40). Our results also clearly indicated functional disturbance in speech processing circuits in patients with AVHs.

Functional alterations in Broca’s and Wernicke’s areas in patients with schizophrenia who experience AVHs have been widely reported in fMRI studies (4). However, there have been few reports of abnormal resting FC between these areas, and most results have been inconsistent. Vercammen et al. reported reduced FC between the left temporal-parietal junction and the right homolog of Broca’s area in schizophrenia patients with AVH. However, patients without AVH were not studied, and symptom correlations for hallucination severity were not presented (23). Hoffman et al. found greater FC between the bilateral Wernicke’s area and Brodmann area 45/46 of IFG (Broca’s area) in patients with AVH compared to patients without AVH (41); follow-up analysis indicated greater FC along a loop linking Wernicke’s area, the IFG, and the putamen compared with patients without AVH and healthy controls. In addition, structural connectivity in the left arcuate fasciculus, which connects Broca’s and Wernicke’s areas, was lower in patients with schizophrenia with AVH than in patients with non-AVH schizophrenia and healthy controls (42–45). These results also suggested disrupted functional interactions within speech-processing systems.

AVH have been linked to several cognitive mechanisms, including misattribution or impaired self-monitoring during speech generation, which could be due to disrupted connectivity between frontal and temporo-parietal brain regions (46). Misattribution models of AVH posit that auditory hallucination experiences occur because of failure to monitor internal speech and attributing it to an external source (47, 48), and therefore predict abnormal FC between typical speech processing areas, mainly in the left fronto-temporal network (including Broca’s and Wernicke’s areas), possibly extending to right hemisphere language homolog areas (49). The delayed “corollary discharge” theory (50, 51) proposes that the failure of neural signal transmission between temporal speech perception areas and inferior frontal speech production areas cause precognition disability in inner speech generation, and temporal speech perception areas cannot suppress response intensity in auditory perception areas, which subsequently incorrectly identify inner speech as external speech. However, the directional information of the connection between Broca’s and Wernicke’s areas is not described clearly.

Ćurčić-Blake et al. (7) primarily investigated EC in the language circuitry by using dynamic causal modeling in schizophrenia patients with and without AVH in an inner speech task. Their results showed diminished connectivity from Wernicke’s area to Broca’s area and a decreasing trend in connectivity from homologs of Broca’s and Wernicke’s areas to Broca’s area. Our results were obtained in a resting state by using GCA, but also suggested reduced information transmission from temporal to frontal language areas in schizophrenia AVH patients. Thus, neuronal activity of frontal language areas was less restrained by temporal language areas, leading to diminished self-monitoring and subsequent misperception of internal speech. These findings add to the aforementioned theoretical models from the perspective of information flow. However, similar research methods and results have not been reported and the connection changes in language processing mode underlying AVH have not been fully characterized. Further studies to verify these preliminary findings should be conducted.

### The Memory Circuit

Increased GC from the left hippocampus to left Broca’s area and decreased GC from the right STG to the right hippocampus were observed in this study. The hippocampus is associated with complex memories in humans, and may be a temporary storage site for memories (52). Hippocampal damage was associated with the occurrence of auditory hallucinations in animal models of schizophrenia (53). Several neuroimaging studies have suggested that the hippocampus is involved in auditory hallucinations. Amad et al. (54) showed that functional connections, white matter connections, and hippocampal volume changes were associated with auditory hallucinations. A meta-analysis based on “activation study” demonstrated increased activity in the left hippocampus/parahippocampal region during AVH in patients with schizophrenia (10). This region also connects widely distributed association cortices, including the language areas responsible for hallucinatory experiences. The hippocampus/parahippocampal region could therefore trigger auditory hallucination-related language brain areas, as our findings also suggest.

Based on a resting-state study, some researchers (8) found reduced FC between the left STG and left hippocampus in patients with chronic schizophrenia with auditory hallucinations compared to patients with non-auditory hallucination, which was negatively correlated with the severity of AVH. Baojuan et al. (36) reported a weakened effective connection from the auditory cortex to the hippocampus in patients with schizophrenia with AVH, by using the dynamic causal model, which was consistent with the results of this study. Although no projection fibers have been found between the auditory cortex and hippocampus anatomically, the results of this study and previous studies all suggest that there might be functional interactions between the two regions. However, there is still no clear understanding of how the reduction of the auditory cortical-hippocampal connection is related with the occurrence of auditory hallucinations. Baojuan et al. (36) speculated that since there are long longitudinal fibers (inferior longitudinal fasciculus) linking the visual region and the hippocampus (55), there might be a similar “auditory cortical-hippocampal projection” participating in the processing of auditory information. We hypothesized that reduced auditory cortical-hippocampal connectivity may lead to aberrant memory retrieval, which is regulated by the hippocampus/parahippocampal gyrus, subsequently triggering memory pieces stored in subcortical regions, especially those related to language, thereby causing the appearance of unconscious auditory hallucinations.

### Limitations

There were some limitations associated with this study. First, the sample size was small, and the study only included patients from one hospital, which might limit the scope and statistical power of our findings. Second, ROI selection was mainly based on previous literature researches, which were frequently reported regions associated with auditory hallucinations. However, several other regions associated with auditory hallucination, but with no clear consensus, were not included, which might limit the exploration of alterations in effective connections. Third, GCA ignores the influence of neurohemodynamics, which may cause displacement distortion and therefore false causality. The dynamic causal model can quantify changes of the effective connection at the neuron level, which can make up for the defects of the Granger causality model but is directly affected by the accuracy of selected ROIs. Therefore, combining data-driven and model-driven analysis by using these two effective connection methods could provide more accurate results and is a promising avenue for future research.

## Conclusion

In this effective connection study based on GCA, we found abnormal connections in specific directions involving the auditory cortex, auditory language formation regions, and hippocampus in schizophrenia with auditory hallucinations, which may explain the mechanism of auditory hallucination from insights of auditory processing, origin of internal speech and memory. These results also provide new imaging evidences in related neural mechanisms of auditory hallucinations from the direction of the information flow.

## Data Availability Statement

The raw data supporting the conclusions of this article will be made available by the authors, without undue reservation.

## Ethics Statement

The studies involving human participants were reviewed and approved by the Ethics Committee of Shaanxi Provincial People’s Hospital. The patients/participants provided their written informed consent to participate in this study.

## Author Contributions

XiaZ and JY were responsible for the study concept and design. JG, DZ, and LW carried out the literature search and analyzed the data. JG wrote the first draft of the manuscript. YF, YW, and XinZ carried out the image acquisition. WW examined patients with psychopathological scales. JG, DZ, MT, and XL assisted with data analysis and interpretation of findings. All authors contributed to the article and approved the submitted version.

## Funding

This work was supported by National Natural Science Foundation of China (No. 81801327).

## Conflict of Interest

The authors declare that the research was conducted in the absence of any commercial or financial relationships that could be construed as a potential conflict of interest.
